# Predicting Abnormalities in Laboratory Values of Patients in the Intensive Care Unit Using Different Deep Learning Models: Comparative Study

**DOI:** 10.2196/37658

**Published:** 2022-08-24

**Authors:** Ahmad Ayad, Ahmed Hallawa, Arne Peine, Lukas Martin, Lejla Begic Fazlic, Guido Dartmann, Gernot Marx, Anke Schmeink

**Affiliations:** 1 Chair of Information Theory and Data Analytics Rheinisch-Westfälische Technische Hochschule Aachen Aachen Germany; 2 Department of Intensive Care and Intermediate Care University Hospital Rheinisch-Westfälische Technische Hochschule Aachen Aachen Germany; 3 Fachbereich Umweltplanung/Umwelttechnik - Fachrichtung Informatik Trier University of Applied Sciences Trier Germany

**Keywords:** anomaly detection, DNN, time series classification, lab values, ICU, CNN, medical Informatics, EHR, machine learning, lightGBM

## Abstract

**Background:**

In recent years, the volume of medical knowledge and health data has increased rapidly. For example, the increased availability of electronic health records (EHRs) provides accurate, up-to-date, and complete information about patients at the point of care and enables medical staff to have quick access to patient records for more coordinated and efficient care. With this increase in knowledge, the complexity of accurate, evidence-based medicine tends to grow all the time. Health care workers must deal with an increasing amount of data and documentation. Meanwhile, relevant patient data are frequently overshadowed by a layer of less relevant data, causing medical staff to often miss important values or abnormal trends and their importance to the progression of the patient’s case.

**Objective:**

The goal of this work is to analyze the current laboratory results for patients in the intensive care unit (ICU) and classify which of these lab values could be abnormal the next time the test is done. Detecting near-future abnormalities can be useful to support clinicians in their decision-making process in the ICU by drawing their attention to the important values and focus on future lab testing, saving them both time and money. Additionally, it will give doctors more time to spend with patients, rather than skimming through a long list of lab values.

**Methods:**

We used Structured Query Language to extract 25 lab values for mechanically ventilated patients in the ICU from the MIMIC-III and eICU data sets. Additionally, we applied time-windowed sampling and holding, and a support vector machine to fill in the missing values in the sparse time series, as well as the Tukey range to detect and delete anomalies. Then, we used the data to train 4 deep learning models for time series classification, as well as a gradient boosting–based algorithm and compared their performance on both data sets.

**Results:**

The models tested in this work (deep neural networks and gradient boosting), combined with the preprocessing pipeline, achieved an accuracy of at least 80% on the multilabel classification task. Moreover, the model based on the multiple convolutional neural network outperformed the other algorithms on both data sets, with the accuracy exceeding 89%.

**Conclusions:**

In this work, we show that using machine learning and deep neural networks to predict near-future abnormalities in lab values can achieve satisfactory results. Our system was trained, validated, and tested on 2 well-known data sets to ensure that our system bridged the reality gap as much as possible. Finally, the model can be used in combination with our preprocessing pipeline on real-life EHRs to improve patients’ diagnosis and treatment.

## Introduction

### Background

Machine learning and data analysis methods are used for diverse applications, such as anomaly detection [1], text classification [2], image segmentation [3], and time series forecasting [4]. One of the fields in which machine learning has become extremely popular recently is medicine. In medicine, there are now other application due to the improved availability of data. In particular, medical images [5] and electronic health records (EHRs) [6,7] represent prominent examples here. Much research has been done on medical images to detect diseases, such as pneumonia [8], which was driven by the advancements in computer vision. In addition, EHRs enabled the use of machine learning models to perform many tasks, such as predicting hospital length of stay [9] and mortality in septic patients [10]. In these studies, the authors used EHRs to train their machine learning models. However, EHRs have so much more data that with the right tools, they can support many valuable applications.

In this study, we consider the treatment of critically ill patients in the intensive care unit (ICU). Throughout the treatment of these patients, laboratory data are regularly gathered. Due to the substantial number of values to be monitored in the ICU, which sometimes can be more than 100 lab tests [11], important anomalies or trends may not be noticed. This can lead to suboptimal treatment strategies and complications in the patient’s case. For example, early changes in lab values for patients with COVID-19 are important predictors of mortality [12]. The correct analysis of laboratory anomalies can direct treatment strategies, particularly in the early detection of potentially life-threatening cases. This should aid in resource allocation and save lives by allowing for timely intervention. Furthermore, health care workers spend 30%-50% of their time in front of computers and must deal with a mass of patient data [13,14]. Any savings in that time can free them to spend more time with patients.

### Prior Work

Because of the recent availability of big data in the medical field, especially EHRs, there has been a growing interest in applying machine learning tools for medical applications. Working with medical data from EHRs can be quite challenging due to the inconsistent sampling of lab measurements, high frequency of missing values, and presence of noisy data. Additionally, there is no standardized way to process medical data before applying machine learning algorithms on them. Nevertheless, many authors have managed to process the data and apply machine learning algorithms for medical sequence modeling. Authors [15] have developed a masked, self-attention mechanism that uses positional encoding and dense interpolation strategies for incorporating temporal order. The authors trained and tested their model on the MIMIC-III data set and achieved better performance on them compared to recurrent neural networks (RNNs). The benchmarking tasks include predicting mortality (classification), length of stay (regression), phenotyping (multilabel classification), and decompensation (time series classification) [16]. Although the benchmarking tasks include a classification task, none of these tasks include lab values or the modeling of irregularly sampled sequences with large amounts of sparse data. The benchmark is created to compare different machine learning models on a specific type of medical data extracted from the MIMIC-III data set and covers only cover only 4 tasks. However, MIMIC-III has much more data that can allow for performing many more tasks like the one in this study.

There has also been some work that compares different approaches and machine learning algorithms for learning from irregularly sampled time series, which is mostly the case in medicine. For example, authors [17] compare modeling primitives that allow learning from the different forms of irregular time series, such as discretization, interpolation, recurrence, attention, and structural invariance. The authors discuss the pros and cons of each of these modeling primitives and the tasks for which they are suited. Another study [18] used a recurrence-based approach using specific versions of RNNs called gated recurrent units (GRUs) and discussed the advantages of using it instead of the other approaches. Additionally, authors [19] have proposed a system for early detection of sepsis using an interpolation-based method for data imputation followed by using temporal convolutional networks (TCNs) and dynamic time warping. The authors used a multitask gaussian process for multichannel data imputation and later used a TCN model to predict the probability of a sepsis diagnosis in the future. The authors proved that their proposed algorithm outperforms the state-of-the-art algorithm for sepsis detection. In contrast, we use a discretization-based approach followed by data imputation to convert the irregularly sampled time series to a regularly sampled one, as it provides an easy way to understand, debug, and implement a framework to deal with sensitive lab values that can be generalized effectively to other EHRs.

### Goal of This Study

This work’s objective is to analyze laboratory results (lab values) of patients in the ICU and classify which of these lab values are predicted to be out of the normal range soon (the next time these tests are done) and which are predicted to be normal. This allows health workers to focus on these laboratory values, their significance, their relation to the patient's current case, and their impact on the patient's future condition. This can potentially lead to reducing the length of the ICU stay and mortality [20]. Moreover, health care workers can focus future testing on these lab values and not waste time and resources on unnecessary tests that constitute approximately 50% of the tests ordered in the ICU [21]. Finally, it will allow the medical staff to reduce the time they need to check all the lab values and focus on the relevant ones, giving them more time to spend with patients [14].

## Methods

### Problem Definition

**Figure 1:**
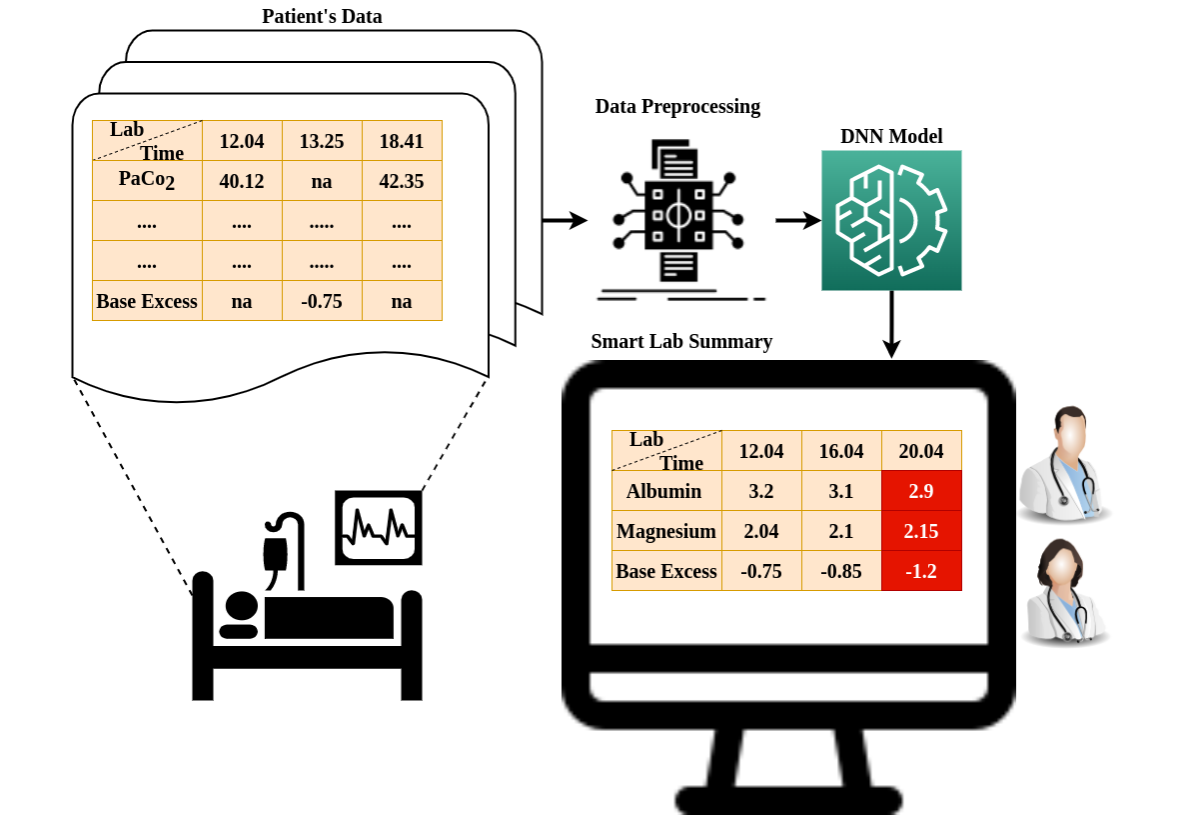
Overall abnormality detection system in practice. DNN: deep neural network.

The task at hand is to predict which lab values will be normal and which will be abnormal in future, for a given period of ICU stay. The input data contain the patients' demographics and numerical lab values from the moment they were admitted till the end of their stay. The output is a binary vector, where each number represents the likelihood of a specific lab value to be abnormal (1) or normal (0) in the next 4 hours. Therefore, our problem is a “many to one” or a multilabel classification problem. Moreover, we have chosen the 4-hour time window because the majority of lab values found in MIMIC-III and eICU are recorded every 4 hours. Therefore, using this time step will introduce the least amount of data artifacts, especially considering that the changes in lab values are not noticeable for smaller time frames (like 1 hour). The same time window for lab values has been used by other authors [22]. Finally, the general diagram of the system is shown in Figure 1.

### Data and Cohort Definition

The data used to train, validate, and test the different prediction models are derived from the MIMIC-III database. It is a database that contains data from 31,532 unique ICU stays of patients who stayed within the ICUs at the Beth Israel Deaconess Medical Center [6] between 2001 and 2012. We also used data derived from the eICU Collaborative Research Database [7]. It is a multicenter database for critical care research created by The Philips eICU program. It contains data on 200,859 ICU stays from 335 ICUs units in the United States of America. In both databases, a unique ICU stay ID is associated with every unique ICU admission.

Our cohort focuses on mechanically ventilated patients in the ICU. This cohort is truly relevant these days because of the COVID-19 virus that caused a sharp increase in the number of patients in the ICU receiving mechanical ventilation. For these patients, it is vital to know which set of lab values have abnormal trends and focus on them, as it has a direct relation to how the case will develop [12]. The same cohort was used in a previous work focused on dynamically optimizing mechanical ventilation in critical care using reinforcement learning [22]. Using this cohort, we extracted 25,086 eICU and 11,943 MIMIC-III ICU stays with mechanical ventilation events. The duration of the ICU patients' stays ranges from 12 h to 72 h in 4-hour time steps. Patient demographics and clinical characteristics are shown in Table 1.

The input data consist of 3 demographic features (age, sex, weight) and 25 lab values (white blood cell count, PaCO_2_, hemoglobin, etc). The lab values chosen are the most relevant to the mechanically ventilated patients, as shown by the medical team members from the university hospital of Rheinisch Westfälische Technische Hochschule (RWTH) Aachen in their previous work [22]. In Multimedia Appendix 1, the chosen features from the MIMIC-III and eICU data sets are listed along with their means and SDs.

The output is a binary vector of length 25. To convert numerical lab values to binary values, we used the reference ranges followed by the American College of Physicians [23]. Finally, the queries of Structured Query Language (SQL) used to extract the cohort data from both databases are included in the Git repository [24].

**Table 1:** Clinical and demographic properties of the study population [16].

Property	MIMIC-III data set	eICU data set
Number of ICUs^a^	5	335
Data acquisition timespan	2001-2012	2014-2015
Number of included patients (N)	11,443	23,699
Age (years), median (IQR)	66.9 (56.3-77.5)	65.0 (54-74)
Body weight in kg, mean (SD)	85.7 (18.1)	83.5 (22.0)
Sex, female, n(%)	4329 (36.3%)	10,546 (42%)
Sex, male, n (%)	7614 (63.7%)	14,540 (58%)
In-hospital mortality, %	11.1	13.2
LOS^b^ in ICU (days), median (IQR)	3.1 (1.6-6.1)	3.0 (1.71-5.9)

^a^ICU: intensive care unit.

^b^LOS: length of stay.

### Preprocessing

The patients’ raw data extracted from the MIMIC-III and eICU data sets were very sparse and had several missing values. Therefore, it was necessary to perform preprocessing to prepare the data for the machine learning pipeline. First, the time-windowed sample-and-hold method was used to handle missing values. In this method, the data sample is held (repeated) until the next available data sample or the maximum hold time is reached. For each feature, we conducted a frequency analysis to determine how often a new measurement is produced. The counts of consecutive measurement time differences are obtained and when their cumulative sum exceeds a threshold, the first value where this occurs is taken as the hold time. When the feature's hold time exceeds this maximum, the data point is considered corrupted [25]. For the rest of the missing values, a k-nearest neighbor imputation with singular value decomposition and mean imputation were used [26]. Any ICU stay that had more than 50% missing data was discarded (occurrence <1% in the overall cohort) [22]. Finally, the Tukey range test was used to detect and delete outliers. The preprocessing steps are explained in detail in the Git repository [24].

### Prediction System Overview

**Figure 2:**
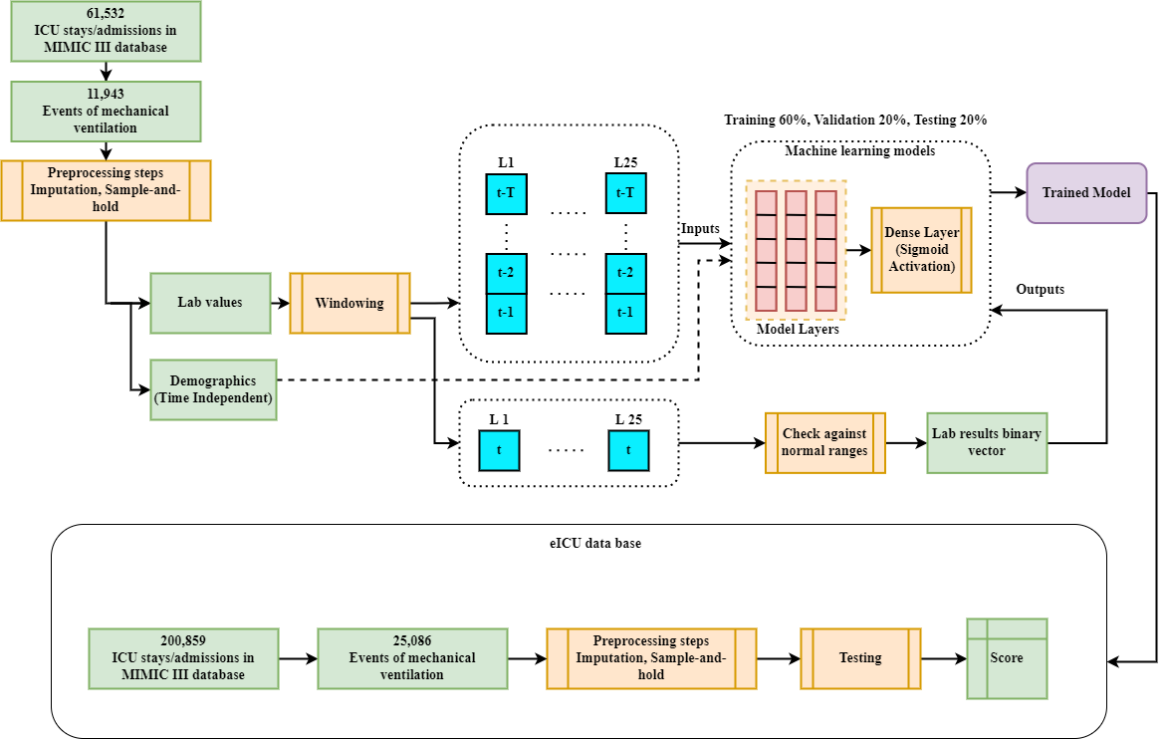
Overall system model used in our study when trained on the MIMIC-III data set and tested on the eICU data set. ICU: intensive care unit; Sigmoid is an activation function; L: lab value; t: time step.

**Figure 3:**
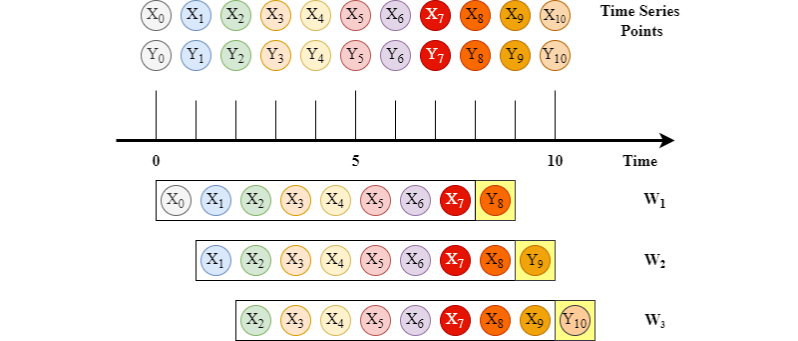
Moving window technique to extract sequences from intensive care unit stays. X and Y represent the input and output data respectively; W represents the windows extracted from the input sequences.

The overall system architecture used for predicting abnormalities in patients' lab values is shown in Figure 2. After performing the preprocessing steps explained earlier, the output time series will be separated into two main types: demographics and lab values. Each ICU stay will be split into multiple shorter sequences using the moving window technique. Figure 3 presents an example of an ICU stay of length L=11 (44 hours). Here, *X_m_* represents the patient's input data vector at time step *m* ∈ 
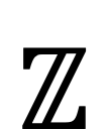
^+^, and *Y_m_* represents the patient's output binary vector. For a window size *W* ∈ 
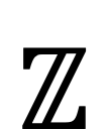
^+^ of 8, we have 3 subsequences extracted from the stay. For example, *W_1_* includes the input vectors [*X_0_:X_7_*] and the output binary vector *Y_8_*. The process of the moving window is applied to ICU stays in the data sets (MIMIC-III, eICU). Then, the resulting subsequences are shuffled and used to train, validate, and test the different machine learning models that we have experimented with, as shown in Figure 2. This means the windowed subsequences from the same ICU stay can be distributed across the training, validation, and testing sets. Moreover, we experimented with different window sizes between *W=5* and *W=10* and chose the one that gave us the best results for all the models, as explained in the Results section.

We experimented with predicting the exact numerical lab values (regression problem) and then converting the predicted output to a binary vector after comparing the values with the normal ranges. The models were then trained to minimize the minimum squared error loss. The results were 10%-20% worse than those obtained when predicting the output binary vector directly and optimizing for the binary cross-entropy loss. Therefore, we selected this system model.

### Prediction Models

The goal of the prediction model in our scenario is to predict abnormalities in laboratory values for a given input sequence. The machine learning problem is a multilabel classification problem because multiple lab values are classified as normal or abnormal at the same time (multiclass) and more than 1 lab value can be abnormal at the same time (multilabel). We experimented with four current deep learning (DL) approaches: long short-term memory (LSTM), self-attention with time encoding (transformer architecture), convolutional neural network (CNN), and TCN. In the following subsections, each model architecture is discussed briefly. The models are explained in more detail in Multimedia Appendix 2 [2,27-39].

#### LSTM models

**Figure 4:**
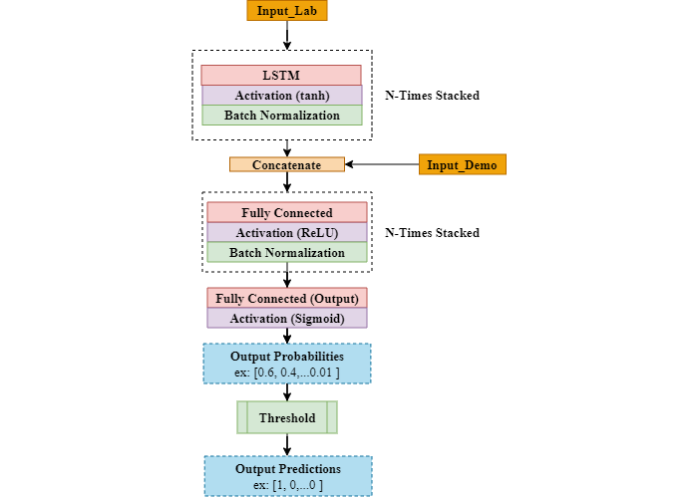
LSTM architecture used in our experiments. LSTM: long short-term memory; ReLU: rectified linear unit; Tanh, ReLU and Sigmoid are activation functions.

LSTM is a type of RNN that has the ability to learn from long sequences of data. A typical LSTM layer in a DL model consists of multiple LSTM cells. Another similar yet simpler cell structure is called GRU [4]. We experimented with both cell types in our model and chose LSTM because it performed better. The architecture used in our experiment is shown in Figure 4. All the lab values will be input to the LSTM block to learn from the sequential data. Each LSTM block includes an LSTM layer, which has “tanh” as the built-in activation function. Then comes a batch normalization layer after the sequential data pass through the layers, and these data will be concatenated with the demographic features. The concatenated data will then go through a stack of fully connected layers ending with a last dense layer that has a sigmoid activation function. During forward propagation, the output probabilities will be compared to a threshold to produce the binary labels that are used to calculate the loss and other evaluation metrics.

#### CNN models

CNNs learn to optimize their kernels to extract information from input data in a successive manner. Additionally, they work well on time series forecasting and classification problems [27], often outperforming LSTMs in terms of the total training time in a more computationally efficient manner [28]. In our case, we used a 1D multiple CNN (M-CNN), where the kernels (filters) move along the time axis performing convolution operations on all features. The kernel size defines how many time steps 1 kernel covers at any point in time.

**Figure 5:**
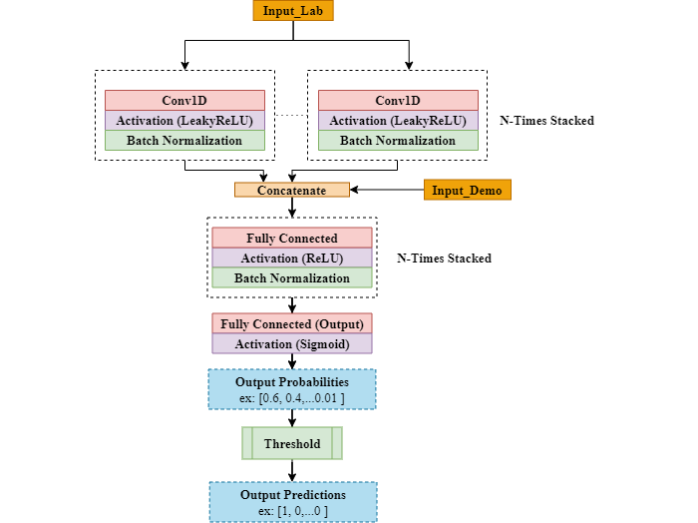
Multiple convolutional neural network model architecture used in our experiments. Conv1D: 1D convolutional layer; LeakyReLU: leaky rectified linear unit; ReLU: rectified linear unit; Sigmoid, LeakyReLU, and ReLU are activation functions.

Aside from the normal CNN that takes 1 input stream, we developed an architecture that takes 2 streams of the input sequences in parallel. Each stream will be processed with different filters. This ensures that we capture short-term dependencies in the sequences as well as long-term ones. The network architecture is shown in Figure 5.

#### Transformer models

Transformers are a recent neural network architecture derived from the attention mechanism first proposed in an earlier study [29]. The mechanism was designed initially for translation tasks, which were earlier accomplished using RNNs.

**Figure 6:**
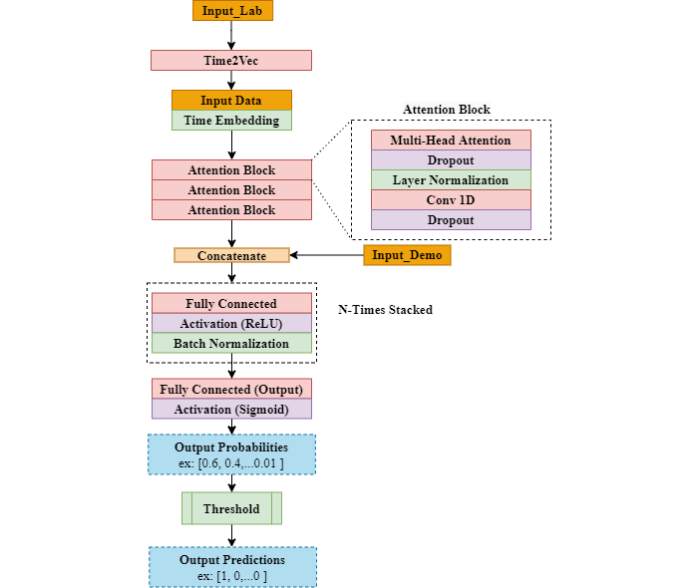
Transformer architecture used in our experiments. Conv1D: 1D convolutional layer; Time2Vec: time to vector transformation; ReLU: rectified linear unit; ReLU and Sigmoid are activation functions.

Transformers typically use a collection of superimposed sinusoidal functions to represent the position of words in natural language processing tasks. However, in time series tasks, we need to attach the meaning of time to our input. Authors [30] have introduced a method where each input feature is represented as a linear component and a periodic component. The result at the end will be a learned vector representation of time steps that will be concatenated with the input data before the attention layers. The model architecture we developed is shown in Figure 6.

#### TCN models

**Figure 7:**
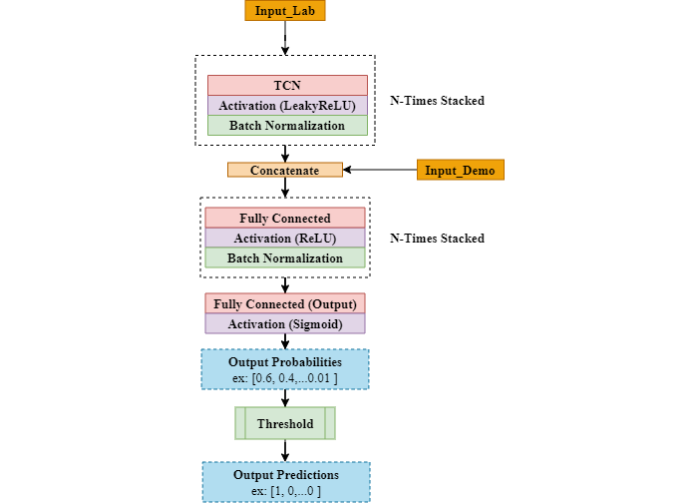
TCN architecture used in our experiments. LeakyReLU: leaky rectified linear unit; ReLU: rectified linear unit; TCN: temporal convolutional network; LeakyReLU, ReLU, and Sigmoid are activation functions.

TCNs were first introduced for video-based action segmentation [31]. Not long after that, they were used for sequence modeling tasks like the detection of sepsis [19]. A TCN differs from a conventional CNN in 2 ways; first, a TCN can take a sequence of any length and output a sequence of the same length using 0 padding; second, a TCN performs causal convolution. In general, TCNs are advantageous because they can be trained in parallel with less memory unlike RNNs. Additionally, they support variable length inputs and can easily replace any existing RNN. Figure 7 shows the TCN architecture that we designed and used in our experiments.

### Evaluation Metrics

In our work, we predicted the output binary vector of the future time step rather than the actual numerical lab values. We tried training the models as regression models predicting the actual numerical values and minimizing the minimum squared error. Then, we converted the predicted numerical output to binary vectors using the recommended ranges. However, we received better results when we treated the models as multilabel, multiclass classifiers predicting the binary vectors directly. Therefore, the evaluation metrics we used are binary accuracy, precision, recall, and F1 score.

### Evaluation Setup

As we were predicting multiple lab values at the same time and all the classes were of equal importance, we used micro-averaging to calculate the accuracy, precision, recall, and F1 globally. These evaluation metrics were used to evaluate the models' training, validation, and testing. Additionally, to compare the models, the following points were followed: First, the models' architectures and hyperparameters were optimized using the Keras Tuner library [40] to ensure that the models performed at their best. Second, the models were trained to optimize the binary cross-entropy loss [41]. Third, early stopping was used to stop the model's training once the validation loss did not change by 0.01 for 10 consecutive epochs. This reduces the chances of model overfitting. Fourth, we set the seed for all the random processes during model training to ensure replicability of our results. Finally, we used the same threshold (TH=0.5) and same window size (sequence length=6) for all the models to ensure a fair comparison. We used 0 padding for sequences shorter than 6 time steps (ICU stay length<24 hours). Moreover, we implemented a gradient boosting–based method (LightGBM) for comparison with DL-based methods. LightGBM is one of the best performing non-DL–based algorithms that is shown to perform well on time series classification tasks [32].

We experimented with 2 approaches for training the models. In the first approach, we trained the models and validated them on the MIMIC-III data set. Then, we tested them on the MIMIC-III and eICU data sets, as shown in Figure 2. In the second approach, we trained and validated them on the eICU data set instead. Then, we tested them on the eICU and MIMIC-III data sets. Table 2 shows counts of the training, validation, and testing samples used in both methods from each data set (window size=6). The same cohort of patients was used in both cases, but eICU has much more patient data that led to a much bigger set than MIMIC-III. Finally, the model architectures and hyperparameters can be found on our Git repository [24] and in Multimedia Appendix 2.

**Table 2:** Sample counts for training, validation, and testing in both training methods.

Method	Number of training samples	Number of validation samples	Number of first testing samples	Number of second testing samples
#1	73,190 (MIMIC-III)	12,915 (MIMIC-III)	21,526 (MIMIC-III)	196,208 (eICU)
#2	166,776 (eICU)	29,431 (eICU)	49,052 (eICU)	86,106 (MIMIC-III)

### Ethics Approval

Approval for data collection, processing, and release for the MIMIC-III database has been granted by the Institutional Review Boards of the Beth Israel Deaconess Medical Center (Boston, United States) and Massachusetts Institute of Technology (Cambridge, United States). Approval for data collection, processing, and release for the eICU database has been granted by the eICU research committee and exempt from Institutional Review Board approval. All data were processed using the computational infrastructure at the RWTH Aachen University and the University Hospital at RWTH Aachen in accordance with European Union data protection laws.

## Results

**Figure 8:**
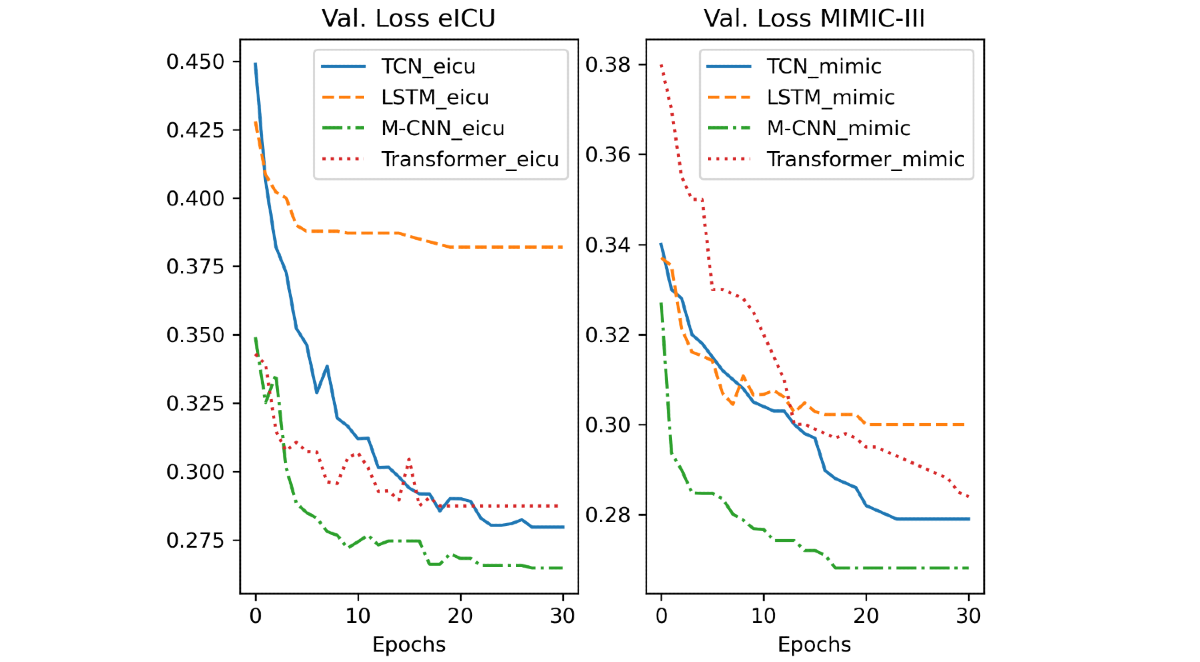
Validation loss of the different models. LSTM: long short-term network; M-CNN: multiple convolutional neural network; TCN: temporal convolutional network; Val.: validation; ICU: intensive care unit.

**Figure 9:**
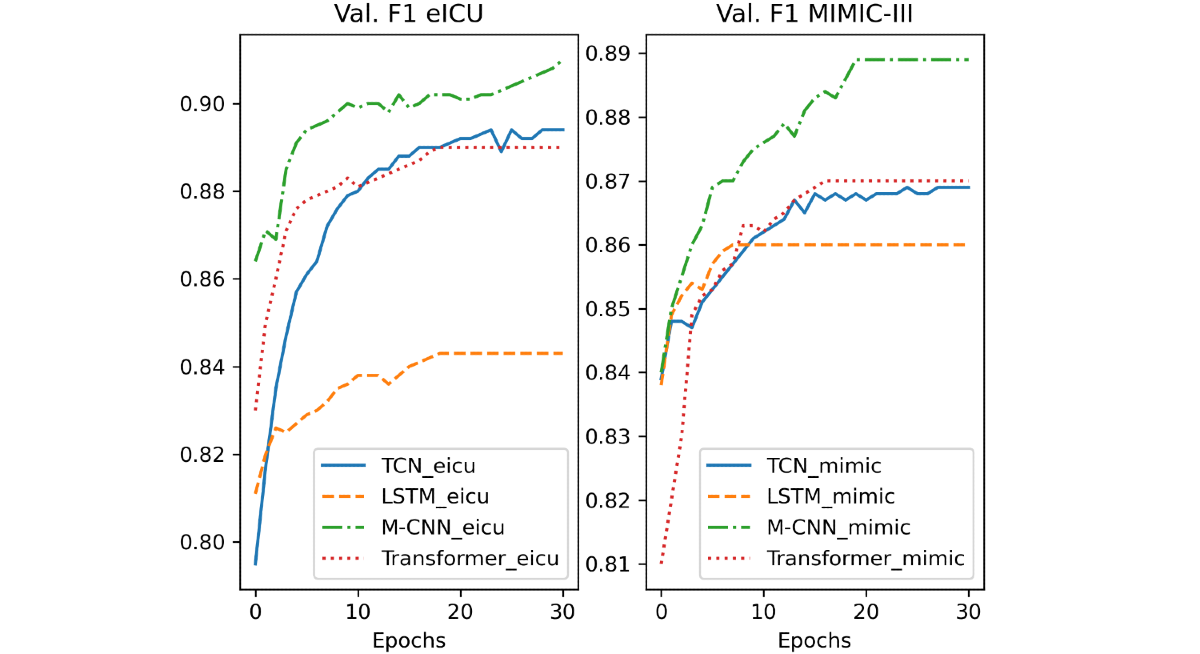
Validation F1 score of the different models. LSTM: long short-term network; M-CNN: multiple convolutional neural network; TCN: temporal convolutional network; Val.: validation; ICU: intensive care unit.

**Figure 10:**
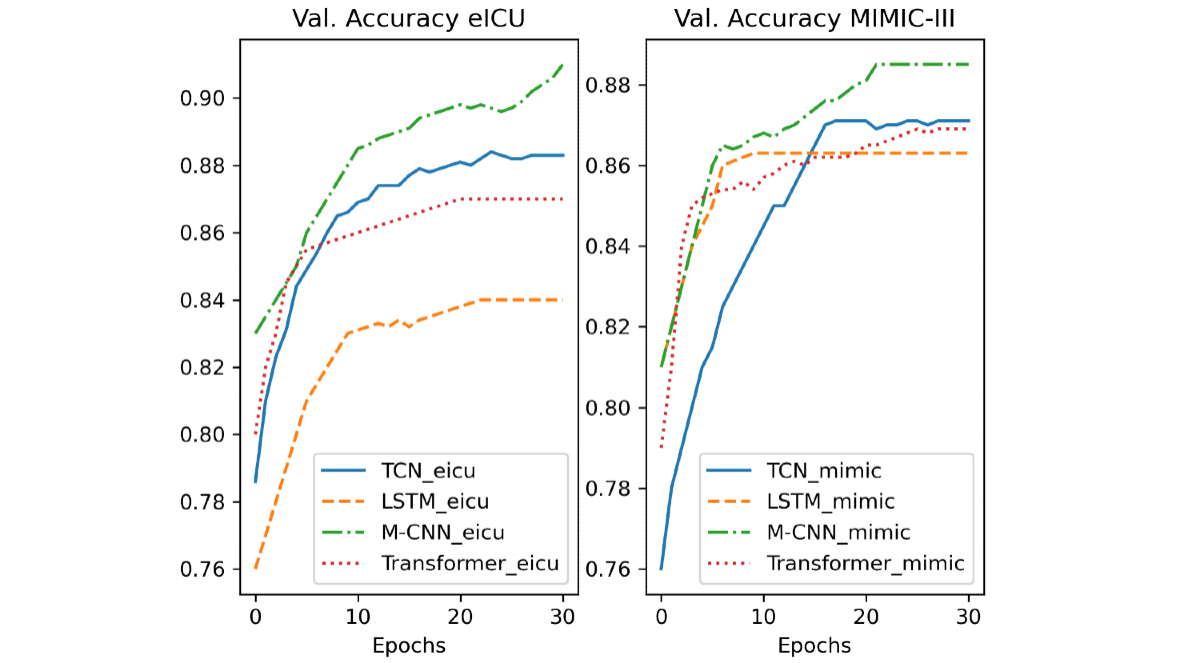
Validation accuracy of the different models. LSTM: long short-term network; M-CNN: multiple convolutional neural network; TCN: temporal convolutional network; Val.: validation; ICU: intensive care unit.

In Figures 8, 9 and 10, we report the validation loss, F1 score, and accuracy of the different models during training, respectively. The models’ names ending with “mimic” indicate that they were trained on the MIMIC-III data set and those ending in “eicu” refer to the models trained on the eICU data set. Moreover, because of the early stopping used during training, some models stopped training before others. Thus, their metrics are constant after the stopping point.

In Tables 3 and 4, we report the testing accuracy, recall, precision, and F1 scores of the different models. All the results were averaged over all the lab values and the testing samples.

As we expect our system to run continuously on huge amounts of data in hospitals, we want the performance of the chosen model to be good enough to meet such demands. Therefore, we measured the models' inference times. Experiments were run on a computer with an Intel(R) Core i9-9900K processor (Intel Corporation) running at 3.60 GHz using a 32-GB DDR4 RAM and Nvidia GTX 1080ti graphics processing unit (Nvidia Corporation), running Ubuntu (version 20.04, Canonical Ltd), Python (version 3.8, Python Software Foundation), and TensorFlow (version 2.6, Google Brain). Table 5 reports the inference time for each model on a whole batch (batch size=128 samples).

**Table 3:** Testing results for the different models over all lab values (micro-average) on the MIMIC-III data set^a^.

Training data set and model		Accuracy	Precision	Recall	F1 score
**MIMIC-III**					
	LSTM^b^	0.85	0.83	0.87	0.85
	CNN^c^	0.86	0.84	0.85	0.84
	M-CNN^d^	0.88	0.87	0.89	0.88
	Transformer	0.86	0.88	0.81	0.84
	TCN^e^	0.86	0.87	0.85	0.86
	LightGBM^f^	0.83	0.82	0.76	0.78
**eICU**					
	LSTM	0.8	0.79	0.81	0.8
	CNN	0.85	0.86	0.83	0.84
	M-CNN	0.87	0.88	0.86	0.87
	Transformer	0.86	0.86	0.84	0.85
	TCN	0.83	0.82	0.84	0.83
	LightGBM	0.82	0.77	0.78	0.77

^a^The models listed under MIMIC-III were trained on the MIMIC-III data set and those under eICU were trained on the eICU data set.

^b^LSTM: long short-term memory.

^c^CNN: convolutional neural network.

^d^M-CNN: multiple convolutional neural network.

^e^TCN: temporal convolutional network.

^f^LightGBM: gradient boosting–based method.

**Table 4:** Testing results for the different models over all lab values (micro-average) on the eICU data set^a^.

Training data set and model		Accuracy	Precision	Recall	F1 score
**MIMIC-III**					
	LSTM^b^	0.79	0.81	0.8	0.8
	CNN^c^	0.78	0.8	0.8	0.8
	M-CNN^d^	0.8	0.8	0.83	0.81
	Transformer	0.75	0.82	0.69	0.75
	TCN^e^	0.71	0.74	0.72	0.73
	LightGBM^f^	0.75	0.78	0.75	0.76
**eICU**					
	LSTM	0.82	0.85	0.83	0.84
	CNN	0.85	0.86	0.83	0.84
	M-CNN	0.89	0.9	0.91	0.9
	Transformer	0.86	0.87	0.88	0.87
	TCN	0.89	0.88	0.89	0.89
	LightGBM	0.82	0.77	0.78	0.77

^a^The models under MIMIC-III were trained on the MIMIC-III data set and those under eICU were trained on the eICU data set.

^b^LSTM: long short-term memory.

^c^CNN: convolutional neural network.

^d^M-CNN: multiple convolutional neural network.

^e^TCN: temporal convolutional network.

^f^LightGBM: gradient boosting–based method.

**Table 5:** Inference time for the different models.

Model name	Average inference time/batch
LSTM^a^	654 ms
CNN^b^	220 ms
M-CNN^c^	285 ms
TCN^d^	854 ms
Transformer	598 ms
LightGBM^e^	121 ms

^a^LSTM: long short-term memory.

^b^CNN: convolutional neural network.

^c^M-CNN: multiple convolutional neural network.

^d^TCN: temporal convolutional network.

^e^LightGBM: gradient boosting–based method.

## Discussion

In this work, we developed an end-to-end system to extract and process lab results from EHRs and applied various machine learning algorithms to determine which lab values will be out of range in the next 4 hours with satisfactory results. This enables medical staff to focus on these lab values that can lead to improvements in overall patient diagnosis and treatment. Additionally, it can help reduce the time and cost wasted on irrelevant lab tests. The following steps were taken to reach this goal: First, we used SQL queries to extract the relevant patient data following our cohort from MIMIC-III and eICU data sets. Second, we used the time-windowed sample-and-hold method alongside k-nearest neighbor imputation with mean imputation and singular value decomposition to fill missing values. Moreover, we used the Tukey range test to detect anomalies and delete them. Third, we experimented with non-DL methods like LightGBM as well as 4 DL algorithms for time series classification. The DL-based method stacks models through mapping and processing functions between the models, using gradient descent or momentum methods to optimize fit. Gradient boosting methods like LightGBM iteratively fit models to error terms and average results within a generalized linear modeling framework using base learner models at each iteration, introducing a penalty term into the base learner models. Finally, we trained and tested our algorithms on 2 of the well-known EHR data sets, MIMIC-III and eICU. Cross-validating our algorithms on these 2 data sets ensures not only a broader performance comparison, but also helps analyze how far the different algorithms can generalize on new unseen data.

A deeper analysis of the training results of the different DL-based models (Figures 8, 9 and 10) revealed that the M-CNN model trained on the eICU data set yielded better results at the end of the training than any other model. Additionally, we can see that the performance of both the TCN and transformer model improved significantly when trained on more data (eICU data set). This can be better understood from the results in Tables 3 and 4. First, the models trained on the eICU data set generalized better on data that they had not seen before from both the data sets. This is because the models had more data to train on, so they could see more variations and cases that they learned. On the other hand, the models trained on the MIMIC-III data set (43% the size of eICU training samples) performed well on the testing samples from MIMIC-III but performed much worse on the testing samples from eICU. Second, the M-CNN model performed the best in terms of almost all the evaluation metrics in both training methods. CNN models perform well on many sequenced modeling tasks, often outperforming RNN architectures like LSTM or GRU. Additionally, CNN-based models have the least number of trainable parameters out of the different DL-based methods and occupy the least memory, making them perform better on data sets with small amounts of training data. On the other hand, standard CNNs can only work with fixed-size inputs and usually focus on data elements that are in immediate proximity due to their static convolutional filter size. However, combining multiple CNN models helps increase the accuracy further by applying convolutions with multiple filter sizes and combining the outputs to give a more robust prediction. Moreover, in our case, we chose a static, relatively short input sequence length, thus mitigating the issue of long, variable length sequences. In case of long, variable length input sequences, a TCN will be a better candidate. A TCN employs techniques like multiple layers of dilated convolutions and padding of input sequences to handle different sequence lengths and detect dependencies between items that are not next to each other but are positioned on different places in a sequence. Furthermore, more complicated architectures like transformers and TCNs with many more trainable parameters would perform better if they had access to more data, which is often an issue in the medical field because of the scarcity of available training data. Therefore, M-CNN architectures are desirable for modeling medical time series data with static lengths and relatively short lengths like lab values requiring relatively smaller training data sets. Moreover, the M-CNN architecture can generalize well on unseen data when trained well, considering integrated measures for reducing overfitting during model training. An interesting fact is that despite not outperforming the M-CNN model, lightGBM performed as well (sometimes better) as some other DL-based approaches while requiring much less training time. Non-DL–based approaches can model problems with much less training data but require hand-crafted features and are very sensitive to outliers and variation in data. Further, removing seasonality is often needed when dealing with time series data. Finally, we can see that the LightGBM model is the fastest in terms of the inference time according to Table 5, followed by the CNN model, which is the fastest among the DL-based models. The M-CNN model, despite outperforming the regular CNN model, is 29% slower in terms of the inference time, which is expected as the model has more parameters.

Overall, our comprehensive analysis shows the advantage of using DL models for classifying future abnormalities in lab values for patients in the ICU. Although we tested our algorithms on 2 of the most used EHR data sets, further testing is needed to assess the performance of the full pipeline on other EHRs, including the preprocessing steps and how well the tuned hyperparameters of the machine learning models will generalize. Nevertheless, we believe this study can help other researchers trying to use machine learning in modeling medical time series problems.

## Appendix

Multimedia Appendix 1 Statistical properties of the input features from MIMIC-III and eICU data sets.

Multimedia Appendix 2 Details of the models used.

## Abbreviations

CNN: convolutional neural network
DL: deep learning
EHR: electronic health record
GRU: gated recurrent unit
ICU: intensive care unit
LSTM: long short-term memory
M-CNN: multiple convolutional neural network
RNN: recurrent neural network
RWTH: Rheinisch Westfälische Technische Hochschule
SQL: Structured Query Language
TCN: temporal convolutional network
