# Supplementary material for: Predicting Abnormalities in Laboratory Values of Patients in the Intensive Care Unit Using Different Deep Learning Models: Comparative Study
Source: JMIR Med Inform. 2022 Aug 24;10(8):e37658. doi: 10.2196/37658 (PMC9453586; doi:10.2196/37658)
Supplement: Multimedia Appendix 1 [file medinform_v10i8e37658_app1.pdf]

TABLE : Statistical properties of the input features from both MIMIC-III and eICU datasets.

Feature name	Mean MIMIC	Std MIMIC	Mean eICU	Std eICU
Gender	0.37	0	0.42	0
Age	65.13	0.03	63.4	0.12
Weight [Kg]	85.8	0.5	83.15	0.44
SpO2 [%]	97.49	1.49	96.9	1.54
Potassium [mEq/L]	4.24	0.27	4.04	0.29
Sodium [mEq/L]	138.72	1.66	139.75	1.99
Chloride [mEq/L]	106.19	2.52	105.62	2.51
Glucose [mg/dL]	129.55	18.12	134.68	23.71
Blood urea nitrogen [mg/dL]	21.61	4.59	25.38	4.71
Creatinine [mg/dL]	1.09	0.21	1.21	0.20
Magnesium [mg/dL]	2.04	0.17	2.00	0.16
Calcium [mEq/L]	8.33	0.34	8.18	0.32
CO2 [mEq/L]	24.75	1.79	25.49	2.03
Total Bilirubin [mg/dL]	0.99	0.33	0.77	0.16
Albumin [g/dL]	3.2	0.26	2.76	0.23
Hemoglobin [g/dl]	10.63	0.81	10.32	0.78
WBC Count [10^9/L]	11.96	2.55	12.08	2.19
Platelets Count [10^9/L]	184.62	35.52	185.86	26.73
Partial Thromboplastin Time [S]	33.86	3.94	39.19	6.09
Prothrombin time [S]	14.64	0.87	15.83	1.14
International Normalized Ratio	1.34	0.12	1.35	0.11
Arterial PH	7.38	0.03	7.38	0.04
PaO2 [mmHg]	91.27	4.75	106.05	20.25
PaCO2 [mmHg]	40.12	3.21	39.91	3.97
Arterial Base Excess [mEq/L]	-0.75	1.55	-0.11	1.88

Lactate [mmol/L]	2.09	0.5	1.97	0.44
Bicarbonate [mEq/L]	23.47	1.61	24.05	1.86
PaO2/FiO2 Ratio	199.93	17.89	266.4	56.15
