# Supplementary material for: Predicting Abnormalities in Laboratory Values of Patients in the Intensive Care Unit Using Different Deep Learning Models: Comparative Study
Source: JMIR Med Inform. 2022 Aug 24;10(8):e37658. doi: 10.2196/37658 (PMC9453586; doi:10.2196/37658)
Supplement: Multimedia Appendix 2 [file medinform_v10i8e37658_app2.pdf]

Models' details:

LSTM:

LSTM is a type of recurrent neural networks that has the ability to learn from long sequences of data while remembering relevant information from past time steps. A typical LSTM layer in a deep learning model consists of multiple LSTM cells. Each cell has four gates to control the flow of information; input gate, output gate, forget gate and the cell state. These gates can learn which data in a sequence is important to keep or throw away. By doing that, it can pass relevant information down the long chain of sequences to make predictions [35]. Another similar, yet simpler cell structure is called GRU. We experimented with both cell types in our model and chose LSTM because it performed better. The architecture we used in our experiment is shown in (Figure 1). All the lab values will be input to the LSTM block to learn from the sequential data. Each LSTM block include a LSTM layer, which has a "tanh" as a built-in activation function. Then comes a Batch Normalization layer, which reduces the internal covariant shift and reduces the dependence of gradients on the scale of the parameters or their initial values ensuring a more stable training. We do not use a Dropout layer in our architecture because we already use batch normalization which offer the same network stabilization without the risk of variance shift [36]. After the sequential data pass through the layers, it will be concatenated with the demographic's features. The concatenated data will then go through a stack of fully connected layers ending with a last dense layer that has a sigmoid activation function. During forward propagation, the output probabilities will be compared to a threshold to produce the binary labels that are used to calculate the loss and the other evaluation metrics. In (Table 1), we show the important hyperparameters used in our architecture.

Table S1. The LSTM model hyperparameters.

**Layer name**	**Layer details**
LSTM_1_1	no_cells= 50
drop_11	Rate= 0.2
LSTM_1_2	no_cells= 100
drop_12	Rate= 0.3
LSTM_1_3	no_cells= 100
drop_13	Rate= 0.3
Dense_2	no_cells = 40
Dense_3	no_cells = 140
DROP_3	Rate= 0.2
Learning rate	0.01

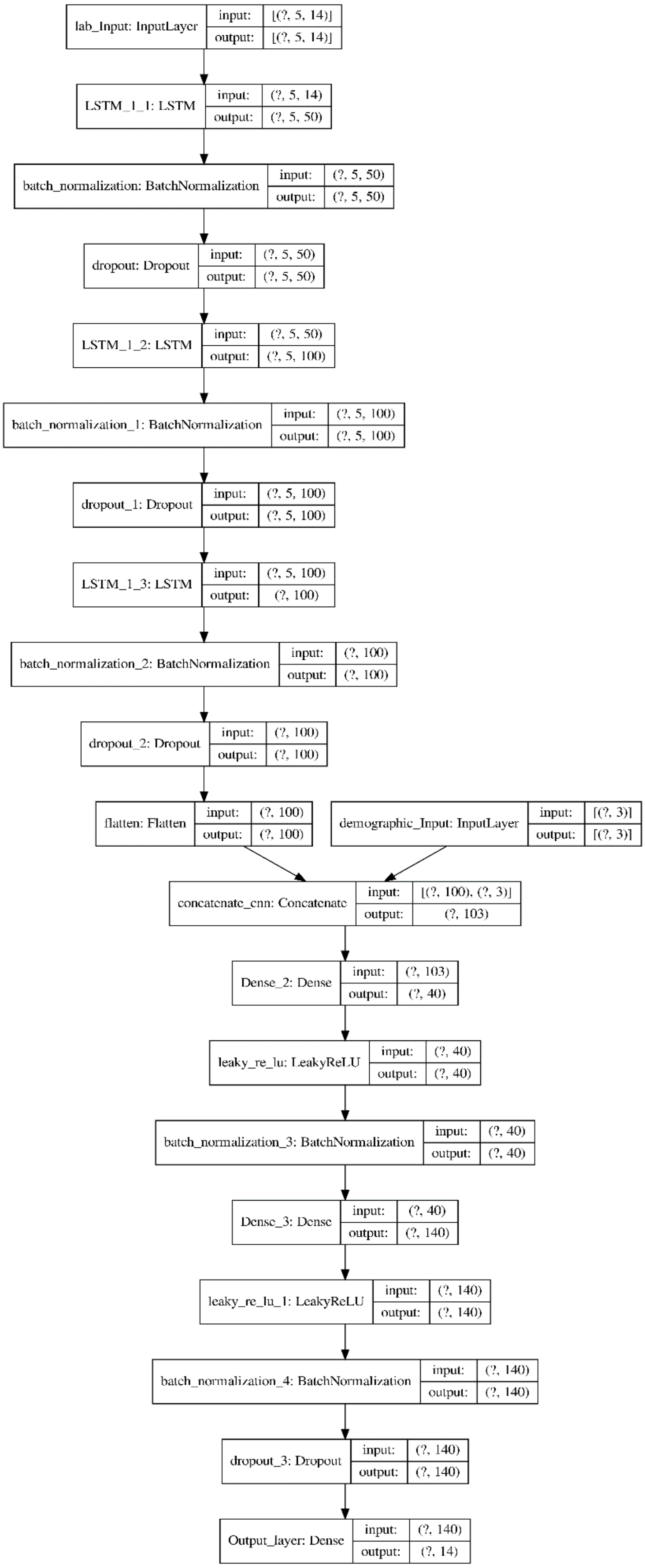

Figure S1. The LSTM model used in our experiments.

CNN

Convolutional neural network is a class of deep neural networks that is inspired by the visual cortex in animals where individual neurons respond to only a specific part of the visual field called receptive field [37]. Convolutional networks learn to optimize its kernels to extract information from input data in a successive manner. CNNs have lots of applications in image classification [38], text classification [2] and natural language processing [39]. Additionally, they proved to work well on time series forecasting problems like in [27], outperforming LSTMs often in terms of total training time in a more computationally efficient manner [28]. In our case we use a 1-Dimensional multi-CNN where the kernels (filters) move along the time axis applying convolution operation on all features. The kernel size defines how many time-steps one kernel covers at any point in time. In (Figure 2), the network architecture used in our experiments is shown and in (Table 2), the important hyperparameters are stated.

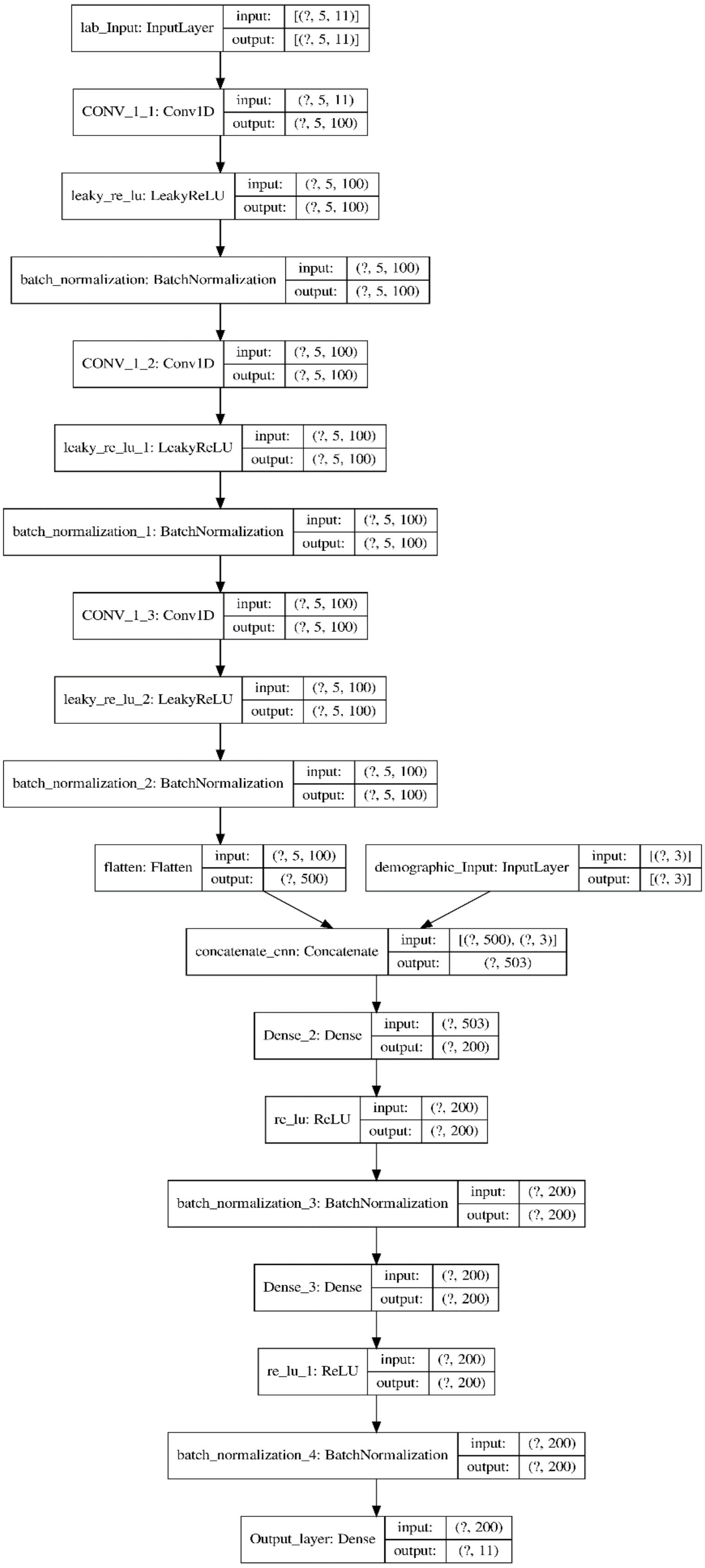

Figure S2. The CNN model used in our experiments.

Table S2. The CNN model hyperparameters.

Layer name	Layer details
CONV_1_1	no_filters= 100, CNN kernel size=2
CONV_1_2	no_filters= 100, CNN kernel size=3
CONV_1_3	no_filters= 100, CNN kernel size=3
Dense_2	no_cells = 200
Dense_3	no_cells = 200
DROP_3	Rate= 0.2
Learning rate	0.0003

Multi-CNN

The CNN architecture we developed takes two streams of the input sequences in parallel. Each stream will be processed with different filters. This ensures that we capture short-term dependencies in the sequences as well as long-term ones. Additionally, in each convolutional block, the activation function is replaced with a modified version of the ReLU activation function called LeakyReLU which performed better than ReLU with CNN. LeakyReLU helps overcome the issue of the "dying ReLU", where the node keeps outputting an activation value of *0* when the summed input to it is negative (it happens with large weights update) [40]. After the convolution blocks, the network continues the same as the LSTM network with fully connected (dense) layers as shown in (Figure 3). In (Table 3), the important hyperparameters for the network are stated.

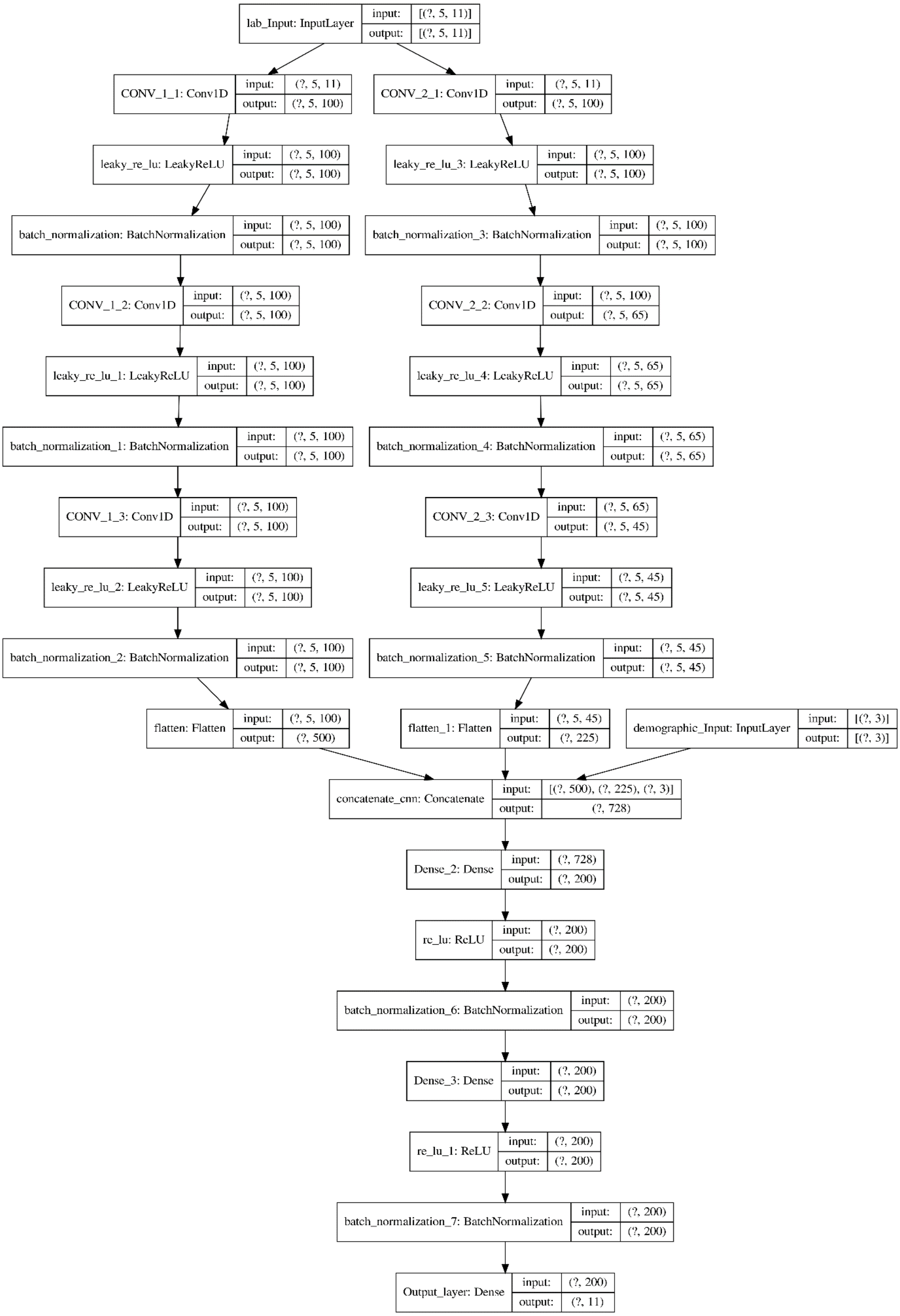

Figure S3. The multi-CNN model used in our experiments.

Table S3. The multi- CNN model hyperparameters.

**Layer name**	**Layer details**
CONV_1_1	no_filters= 100, CNN kernel size=2
CONV_1_2	no_filters= 50, CNN kernel size=3
CONV_1_3	no_filters= 50, CNN kernel size=3
CONV_2_1	no_filters= 100, CNN kernel size=4
CONV_2_2	no_filters= 65, CNN kernel size=3
CONV_2_3	no_filters= 45, CNN kernel size=3
Dense_2	no_cells = 200
Dense_3	no_cells = 200
DROP_3	Rate= 0.2
Learning rate	0.0003

TRANSFORMER

Transformers are a recent neural network architecture that were derived from the attention mechanism first proposed in [29]. The mechanism was designed initially for translation tasks, which was done using RNNs before. Transformers have the advantage that they can be very efficiently parallelized. This allowed training excessively big models on huge datasets. For example, the GPT-3 model has 175 billion parameters and was trained on 45 TB of text data. Additionally, it is quite easy to switch from an existing RNN architecture to a transformer one because inputs are of the same shape. Transformers typically use a collection of superimposed sinusoidal functions to represent the position of words in NLP tasks. However, in time series tasks, we need to attach the meaning of time to our input. One way is introduced by authors in [30], where each input feature is represented as a linear component and a periodic component according to the following equation:

$$t2v(\tau)[i] = \begin{Bmatrix} \omega_i \tau + \varphi_i, & i = 0 \\ F(\omega_i \tau + \varphi_i), & 1 \leq i \leq k \end{Bmatrix}$$

$t2v(\tau)[i]$ is the ith element of $t2v(\tau)[i]$ and (τ) is a scalar notion of time, e.g., days, hours, etc. *F* is a periodic activation function, e.g., the sine function, and φ_i, ω_i are learnable parameters. The result at the end will be a learned vector representation of time steps that will be concatenated with the input data before the attention layers. This model architecture, according to our knowledge, was never used on lab values before. The model hyperparameters we used in our experiments are shown in (Table 4). Each attention block consists of multi-head self-attention, drop out, layer normalization and feed forward layer (1D convolution). After the attention blocks, the architecture continues like the other models.

Table S4. The Transformer model hyperparameters.

Layer name	Layer details
TSTransformer	time2vec_dim = 1, num_attention_heads= 2, head_size=128, num_layers=2, dropout=0
Dense_2	no_cells = 50
Dense_3	no_cells = 100
DROP_3	Rate= 0.3

Learning rate	0.001

TCN

Temporal Convolutional Networks (TCNs) were first introduced in [31] for video-based action segmentation. Not long after that, it was used for sequence modelling tasks like weather prediction [41]. TCN differs from conventional CNN in two ways: First, TCN can take a sequence of any length and output a sequence of the same length using zero padding. Second, TCN perform causal convolution. This means that the output at time *t* is only convolved with samples that occurred before *t*. One of the short comings of the causal convolution, is that the network can look back in time with size linear in the depth of the network. This makes it hard to model long sequences. To overcome this issue, the authors in [31] implemented a version of TCN with dilated convolution that allowed the network to have a large receptive field and to extract long-term patters. The dilated convolution operation ***F*** on element ***s*** of a sequence $x \in R^n$ with a filter $f: 0, \ldots, k-1 \rightarrow R$ is defined by:

$$F(s) = \sum_{i=0}^{k-1} f(i) \cdot x_{s-d \cdot i}$$

where *k* is the filter size, *d* is the dilation factor and $(s - d) \cdot i$ accounts for the direction of the past. In (Figure 4), an example of dilated causal convolution with filter size k=3 and dilation factor *d=1,2,4* is shown. The receptive field can cover all values of the input sequence.

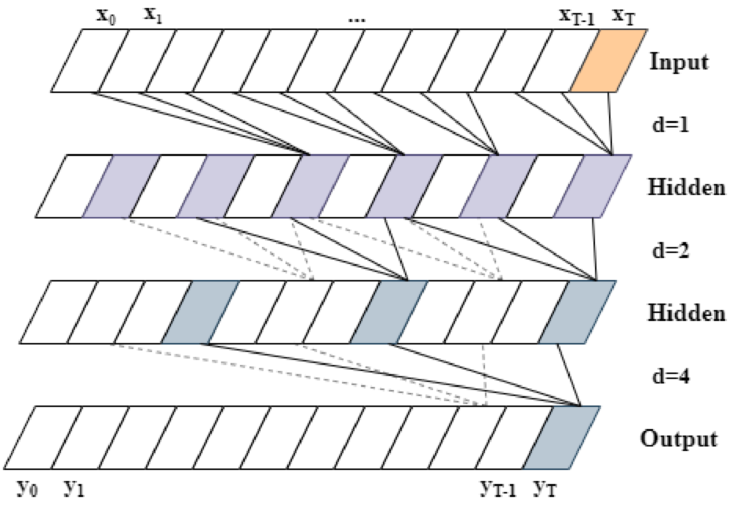

Figure S4. A dilated causal convolution (d=1,2,4, and k = 3).

In general, TCN networks have the advantage that they can be trained in parallel with less memory unlike RNNs. Additionally, they support variable length inputs and can easily replace any existing RNN network. In (Figure 8), the TCN architecture we have designed and experimented with is shown.

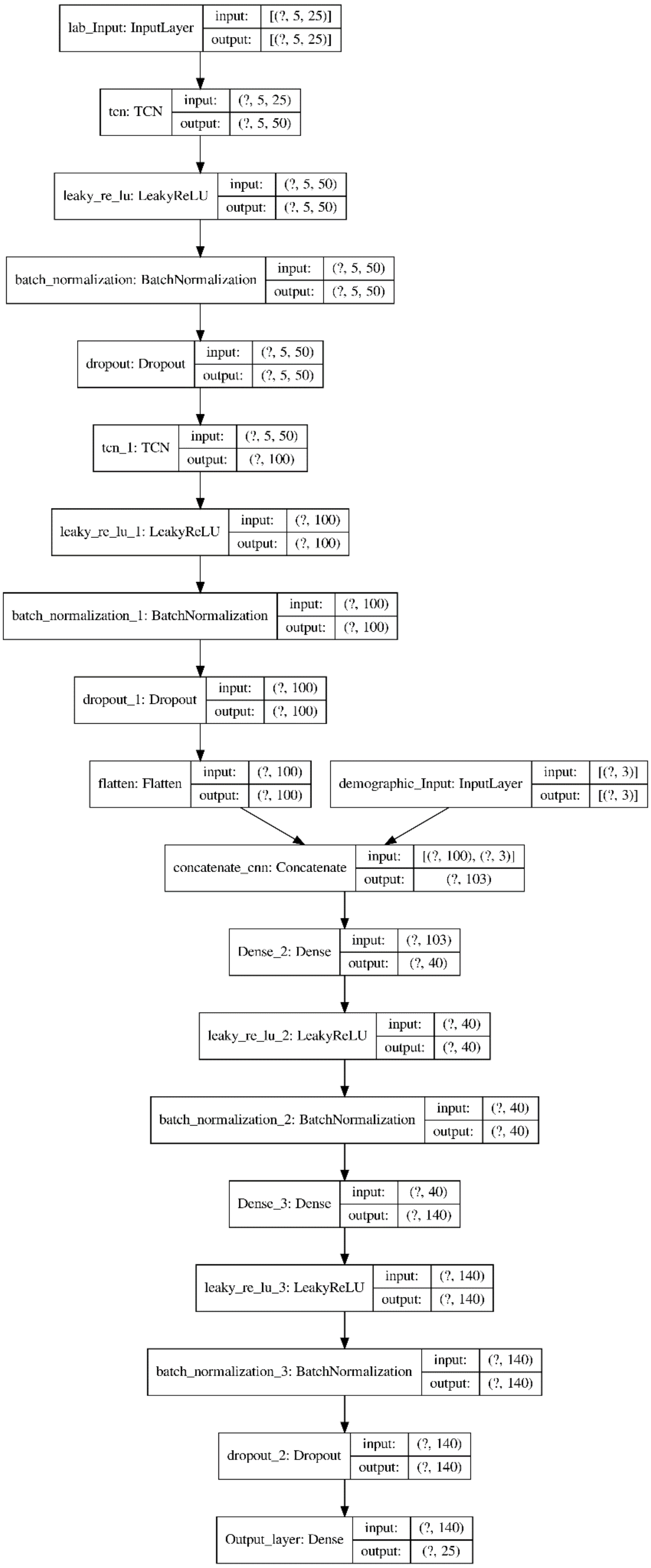

Figure S5. The TCN model used in our experiments.

Table S5. The TCN model hyperparameters.

Layer name	Layer details
TCN_1_1	no_filters= 50, no_stacks= 2
TCN_1_2	no_filters= 100, no_stacks= 2
Dense_2	no_cells = 40
Dense_3	no_cells = 140
DROP_3	Rate= 0.2
Learning rate	0.001

Table 3

LIGHTGBM

We wanted to include a non-deep learning algorithm to be compared with our DL solutions. Therefore, we have picked LightGBM, which is a gradient boosting framework that uses tree-based learning algorithm [34]. We have used the LightGBM python library's implementation of the LightGBM classifier [34]. We have trained the classifier on each of the lab values separately as the classifier does not support the multi-label classification. Then, we tested each trained model on its respected lab value test dataset. Finally, we have used micro averaging to calculate the accuracy, precision, recall and F1 score of the whole dataset.

Layer name	Layer details
LGBMClassifier	learning_rate=0.9, max_depth=-6, random_state=42

Evaluation Metrics

In our work, we are predicting the output binary vector of the future time step rather than the actual numerical lab values. We have tried training the models as regression models predicting the actual numerical values and minimizing the minimum squared error (MSE). Then, we converted the predicted numerical output to binary vectors using the recommended ranges. However, we received better results when we treated the models as multi-label, multi-class classifiers predicting the binary vectors directly. Therefore, the evaluation metrics we used for such classification problems can be derived from (Table 3), which is called the confusion matrix. It is used to evaluate the predictions of a binary classifier by comparing how many outputs were predicted correctly or how many were predicted wrongly to the total number of positive predictions (**P**) or negative predictions (**N**). Additionally, the following evaluation metrics can be derived from the table as follow:

- $Binary\ accuracy = \frac{TP+TN}{P+N}$
- Precision (positive predictive rate) $= \frac{TP}{TP+FP}$
- Recall (true positive rate) $= \frac{TP}{TP+FN}$
- F1 score (harmonic mean) $= 2 \cdot \frac{precision \cdot recall}{precision + recall}$
